# Women entrepreneurship and family-related barriers: an empirical analysis in Southern Benin

**DOI:** 10.3389/fsoc.2026.1873572

**Published:** 2026-08-26

**Authors:** Tchokomi Sabine Toungakouagou Sama, Bruno Montcho, Denga Sahgui

**Affiliations:** 1 Faculty of Arts, Literature and Humanities, University of Parakou, Parakou, Benin; 2 Faculty of Social Sciences, University of Abomey-Calavi, Abomey-Calavi, Benin; 3 Laboratoire Dynamiques Sociales, Éducation, Sport et Développement Humain (LADYS-ES DH), University of Abomey-Calavi, Abomey-Calavi, Benin

**Keywords:** gender intersection, socio-cultural norms, Southern Benin, vulnerability, women's entrepreneurship

## Abstract

This study investigates the family-related and socio-cultural barriers shaping women's engagement in small and medium-sized enterprises in Cotonou and Abomey-Calavi, the main economic hubs of southern Benin. Despite numerous initiatives aimed at promoting women's economic empowerment, institutional support remains insufficient, while entrenched social norms continue to constrain women's entrepreneurial activities. Anchored in the discourse on Sustainable Development Goal 5, the study emphasizes how socio-cultural structures produce and sustain gender-based vulnerabilities. A qualitative research design was adopted, combining a literature review with semi-structured interviews conducted among 43 purposively selected participants, including women entrepreneurs, public and private stakeholders, and representatives of non-governmental organizations. The findings reveal persistent gendered divisions of labor that assign men to productive roles and women to domestic responsibilities, thereby limiting women's entrepreneurial autonomy. These constraints are further differentiated by age: younger women face restricted decision-making power within gerontocratic systems, while adult women encounter pressures linked to marital relations and household authority. Although financial and non-financial support mechanisms, such as microfinance, development programs, and capacity-building initiatives, offer opportunities, women's entrepreneurial trajectories remain significantly constrained. Nevertheless, women demonstrate adaptive strategies to navigate these barriers. However, the social reception of such strategies often reinforces gender stereotypes, particularly when women's economic success challenges established norms. Consequently, many women moderate their entrepreneurial ambitions to maintain social and familial acceptance. The study recommends stronger public policy commitment through the implementation of a comprehensive national gender policy and the integration of gender-sensitive education across all levels. Future research should further examine cultural norms that may serve as levers for women's empowerment in Benin.

## 1 Introduction

In sub-Saharan Africa, women's entrepreneurship has emerged as a critical pathway for both income generation and enhanced social status. Beyond its economic significance, it increasingly reflects women's pursuit of autonomy and empowerment within contexts still marked by structural and socio-cultural constraints. The creation of medium- and large-scale enterprises by women illustrates notable progress, often associated with higher levels of education, professional experience, and the development of managerial and leadership competencies. Such entrepreneurial engagement also suggests improved access to financial resources and the adoption of more formalized and sustainable financial practices. However, despite these advances, women entrepreneurs continue to navigate a complex landscape shaped by family responsibilities, entrenched social norms, gender-based discrimination, and persistent stigma, all of which may limit the scope and sustainability of their entrepreneurial activities.

Women's disproportionate responsibility for unpaid domestic work remains a significant barrier to their participation in paid employment and entrepreneurial activities, as it generates time constraints and perpetuates gender inequalities. Even when women engage in paid work, their earnings are often insufficient to ensure genuine economic independence and autonomy. In sub-Saharan Africa, women's employment is further characterized by persistent wage disparities relative to men, concentration in lower-return sectors (Campos and Gassier, 2017), and a strong presence in the informal economy. Nevertheless, these dynamics are not uniform; variations across political, social, cultural, and symbolic contexts, both within and between developed and developing countries, are critical for a nuanced understanding of women's economic participation.

In sub-Saharan Africa, women's entrepreneurship is widely viewed as a pathway to income generation, social mobility, and autonomy. The growth of women-led enterprises often reflects higher education, professional experience, managerial capacity, and access to financial resources, but these gains remain constrained by family obligations, gender norms, discrimination, and stigma.

Women's unpaid domestic work continues to limit participation in paid employment and business development by reducing available time and reinforcing inequality. Even in paid work, women's earnings are frequently insufficient to secure economic independence, and their labor remains concentrated in lower-return activities and the informal economy (Campos and Gassier, 2017). Although entrepreneurship is associated with job creation, wealth generation, poverty reduction, and national development (Rouatbi and Hernandez, 2017; Rachdi, 2016; Bouzid et al., 2024; Sissoko et al., 2024), comparative studies show that women entrepreneurs face distinct constraints in balancing professional and family responsibilities (Cornet and Constantinidis, 2004; Rouatbi and Hernandez, 2017; Fusulier and Nicole-Drancourt, 2015). Their experiences are often marked by unequal treatment, inequitable conditions, and lower performance outcomes (Brière et al., 2017; Assoumou Menye and Guetsop Sateu, 2018; Johnson and Mehta, 2024), largely because domestic roles remain socially assigned to women (Thakur, 2013).

Female entrepreneurship plays a significant role in Benin's economy. Women represent 40.9% of small business owners, are concentrated in commerce and craft activities, and are strongly represented in the informal sector; 95.3% of women-owned small businesses operate informally, compared with 80% of those owned by men (Institut National de la Statistique et de l'Analyse Économique – INSAE, 2010; Institut National de la Statistique et de la Démographie – INStaD, 2024). Although public policies have increasingly sought to improve the conditions for women's business creation, progress remains uneven. The number of formal women-led enterprises more than doubled between 2019 and 2022, rising from 8,936 to 18,764, indicating a clear but still fragile process of formalization (Institut National de la Statistique et de la Démographie – INStaD, 2024). Women's businesses are also geographically concentrated, especially in urban areas and in the Littoral, followed by Atlantique, Ouémé, the southern region, and Borgou [Institut National de la Statistique et de la Démographie – INStaD, 2024; Chambre de Commerce et d‘Industrie du Bénin (CCIB), and Observatoire du Commerce, de l'Industrie et des Services (OCIS), 2023].

Benin has no law specifically dedicated to female entrepreneurship, but its policy environment includes Act No. 2020–03 on MSME promotion and ratification of CEDAW in 1992. In addition, state- and donor-supported initiatives have promoted women's entrepreneurship through training, technical assistance, and access to credit, notably through the Women Business Promotion Center and the “Microcrédit Alafia” programme, which provides loans without collateral requirements (Glidja, 2019). Despite these measures, institutional support often remains disconnected from women's lived realities, while family and social environments continue to constrain entrepreneurial development.

Prior studies on female entrepreneurship in Benin, Africa, and other contexts have examined women entrepreneurs' profiles, the transfer of entrepreneurial models across countries, support mechanisms, and work-family conflict (Vercruysse and Birkner, 2021; Benhabib et al., 2014; Bouzid et al., 2024; Al-Alawi and El Nagaar, 2018; Doubogan Onibon, 2016; Johnson and Mehta, 2024; Oluwafemi and Duff, 2024; Oyeniran and Lili, 2020; Vampo and Ouattara, 2023; Chergui, 2022). However, this literature often treats women entrepreneurs as a homogeneous category and pays limited attention to how they negotiate empowerment within family and community settings, or to how these negotiations shape business performance. This study addresses that gap by examining how women in Benin initiate and develop small and medium enterprises within persistent sociocultural structures, and by analyzing the strategies they use to manage these constraints.

The study pursues three objectives. First, it identifies the shared and specific vulnerabilities affecting businesses led by adult and young women. Second, it examines opportunities for supporting women's business development. Third, it analyses women's strategies and stakeholders' perceptions of the links between these strategies, business performance, and family dynamics. The article is structured as follows: the first section presents the theoretical framework, the second describes the methodology, the third reports the results, and the fourth discusses the findings.

## 2 Theoretical framework

This research draws on social role theory (Eagly, 1987) and strategic actor theory (Crozier and Friedberg, 1977) to analyse the differentiated vulnerabilities and strategic behaviors of women entrepreneurs in southern Benin. Social role theory explains how socially constructed expectations or roles assign women primary responsibility for domestic and caregiving work and shows how structural inequalities reproduce gender norms across social institutions. A more recent formulation argues that gender beliefs create persistent inequalities that advantage men in leadership and economic roles while devaluing women's authority (Ridgeway, 2011). From this perspective, the intersection of gender and age produces differentiated vulnerabilities, as younger and adult women face stronger constraints due to their lower status and authority within households and communities.

Strategic actor theory conceptualizes women entrepreneurs as strategic actors who negotiate their scope for action within systems of power involving family members, institutional actors, and community norms. This perspective highlights how social vulnerabilities shape the social reception of business support strategies: strategies perceived as transgressive of gender and age norms tend to generate household conflict, whereas conformist strategies are more socially accepted but may constrain business growth. These dynamics give rise to two exclusive entrepreneurial trajectories, in which the pursuit of business performance may undermine family stability, while the preservation of household harmony may limit enterprise expansion.

**Figure 1:**
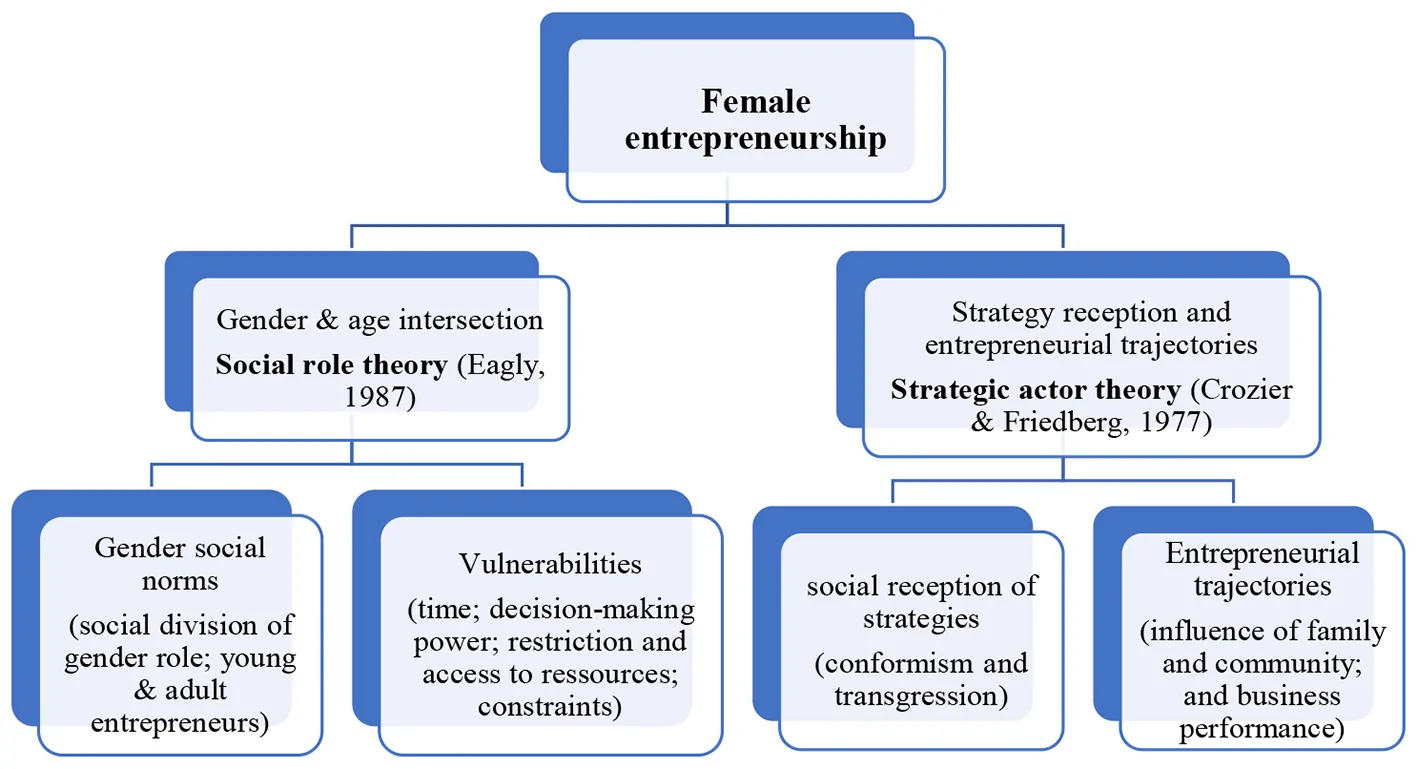
Analytical framework of the research.

Figure 1 presents the analytical framework that combines these two theoretical perspectives. The first variable relates to the intersection of gender and age and uses the concepts of social norms and vulnerability to emphasize the social division of gender roles, disparities between young and adult women, and the factors that contribute to their vulnerability. The second variable identifies business development strategies, distinguishing between those that foster growth and those that generate household conflict according to their level of conformity with social norms. It also examines social actors' positions regarding the relationships between support strategies, business performance and family dynamics, and shows how power relations and normative expectations shape entrepreneurial outcomes.

## 3 Methods

This study adopted a qualitative design structured around the description of the study area, participant selection, data collection, and data analysis.

### 3.1 Description of the research areas

The Republic of Benin, a coastal country in West Africa, provides a pertinent context for examining female entrepreneurship. Despite women's considerable entrepreneurial potential, persistent gender inequalities continue to constrain their access to productive resources and economic opportunities. According to the Ministry of Social Affairs and Microfinance (*Ministère des Affaires Sociales et de la Microfinance*—Ministère des Affaires Sociales et de la Microfinance – MASM, 2019), these structural disparities remain a significant barrier to women's economic participation and entrepreneurial development. Consequently, Benin constitutes an appropriate empirical setting for investigating the factors shaping women's entrepreneurial engagement within a socio-economic environment characterized by enduring gender-based inequalities.

This study focuses on southern Benin, specifically the cities of Cotonou and Abomey-Calavi, the country's two largest urban centers. These cities were selected because they represent the principal hubs of economic activity and population concentration in Benin, providing a suitable context for examining women's entrepreneurship. Cotonou, the country's economic capital, has a population of 679,012, of whom approximately 52% are women (INSAE, 2016). Abomey-Calavi, the second largest city, has a population of 656,358, with women accounting for approximately 51% of its inhabitants (INSAE, 2016). Together, these cities encompass a diverse cross-section of Benin's ethnic groups. Although several languages are spoken, *Fongbe* is the predominant first language, particularly across the southern regions of the country.

### 3.2 Selection of the research participants

Participants were grouped into two categories: resource persons and women entrepreneurs. Resource persons included representatives of public and private organizations, local NGOs, and family members associated with women's partners, selected for their involvement in initiatives supporting female entrepreneurship. Women entrepreneurs were recruited based on three criteria: engagement in small and medium-sized artisanal activities, age (young women aged 18 – 35; adult women aged 36 and above), and location in one of the two study sites. Purposive sampling was used to identify key institutional and organizational actors, followed by snowball sampling to reach women entrepreneurs. Oral informed consent was obtained from all participants. In total, 51 individuals were interviewed: 28 in Cotonou and 23 in Abomey-Calavi.

### 3.3 Data collection methods

To address the research objectives, this study adopted a qualitative research design. Data collection was guided by the research objectives, which informed the identification of the information required to address the research questions. In addition, a systematic literature review was conducted throughout the research process to establish the theoretical and empirical foundations of the study and to inform the development of the interview guide.

Primary data were collected through in-depth, semi-structured interviews with the selected participants. This method was considered particularly appropriate for the exploratory nature of the research, as it facilitated an in-depth understanding of participants' experiences, perceptions, and entrepreneurial trajectories within their social and institutional contexts. Moreover, semi-structured interviews provided the flexibility to explore emergent themes while ensuring consistency across interviews, thereby enabling a nuanced examination of the gender-specific dimensions of entrepreneurship in southern Benin.

#### 3.3.1 Data

The selection of data for this study was guided by two principal considerations. First, the research sought to examine the gender-specific factors shaping women's entrepreneurship, with particular emphasis on the implications of entrepreneurial engagement for family structures, roles, and relationships. Second, it aimed to explore generational differences by examining how younger and older women entrepreneurs differ in their perceptions, motivations, experiences, and entrepreneurial trajectories.

Consistent with these objectives, the study generated qualitative data on women's lived experiences of entrepreneurship and the complex socio-economic, cultural, and institutional factors influencing their entrepreneurial activities (Creswell, 2007). It also examined participants' perceptions of, and experiences with, formal and informal support mechanisms, as well as the perspectives of other relevant stakeholders involved in or influencing women's entrepreneurial activities. Particular attention was paid to the broader social implications of entrepreneurship, especially its effects on family dynamics, gender roles, and the negotiation of domestic and economic responsibilities.

#### 3.3.2 Literature review

To ensure a comprehensive and rigorous review of the literature on women's entrepreneurship, a structured desk-based search strategy was employed. Relevant academic publications were systematically identified through major scholarly databases, including Cairn.info, Google Scholar, and Scopus. The literature search was conducted throughout the research process to incorporate emerging scholarship and to refine the study's conceptual and analytical framework as the research progressed.

A structured review matrix was developed to guide the identification, screening, and analysis of the selected sources. This framework facilitated the systematic extraction, organization, and synthesis of the relevant evidence. The review focused on three interrelated themes: (i) gender inequalities and women's economic participation in Sub-Saharan Africa; (ii) the intersection of women's domestic and entrepreneurial responsibilities within West African contexts; and (iii) the characteristics, opportunities, and constraints of women's entrepreneurship in the region. Particular attention was paid to the structural, institutional, socio-cultural, and economic factors influencing women's entrepreneurial activities, as well as to the barriers, enabling conditions, and outcomes associated with entrepreneurial engagement.

#### 3.3.3 Semi-structure interviews

As noted by Flick et al. (2004, p. 3), “Qualitative research is interested in how people construct the world around them, how they make sense of their lives, and how they interpret their experiences.” Consistent with this interpretivist perspective, primary empirical data were collected through individual semi-structured interviews conducted in southern Benin. This method was selected because it offers sufficient flexibility to explore participants' perspectives in depth while maintaining a consistent framework across interviews. It is particularly well suited to research seeking to understand lived experiences, meanings, and social interactions within specific cultural and institutional contexts.

The interviews were conducted with a diverse range of participants, including women entrepreneurs, male entrepreneurs, representatives of non-governmental organizations involved in entrepreneurship support, and family members of women engaged in entrepreneurial activities. The inclusion of these different stakeholder groups enabled the triangulation of perspectives and provided a more comprehensive understanding of the social, cultural, and relational dimensions of women's entrepreneurship.

An interview guide was developed to ensure consistency across interviews while allowing sufficient flexibility to probe emerging issues and adapt follow-up questions to participants' responses. The guide covered several key themes, including the gender-specific constraints experienced by women entrepreneurs, generational differences between younger and older women, access to entrepreneurial support mechanisms, and the strategies adopted to establish and sustain entrepreneurial activities. Particular attention was also paid to the perceived effects of entrepreneurship on family life, including changes in gender roles, household responsibilities, work-family balance, and interpersonal relationships.

Data collection was undertaken between December 2025 and February 2026. This period coincided with several major festive and cultural events, including Christmas, the New Year celebrations, the National Vodun Festival, and Valentine's Day. For many artisans and small-scale entrepreneurs, these events generate increased demand for goods and services, resulting in heightened commercial activity. Conducting the interviews during this period therefore provided an opportunity to capture participants' entrepreneurial experiences and business practices at a time of intensified economic engagement, thereby enriching the empirical insights generated by the study.

Data collection continued until thematic saturation was reached, that is, the point at which additional interviews no longer generated substantially new themes, concepts, or insights relevant to the research objectives (Guest et al., 2006; Saunders et al., 2018). Saturation was monitored throughout the fieldwork through the iterative analysis of interview transcripts and regular comparisons of emerging themes and items. As the interviews progressed, recurring patterns became increasingly evident across participant groups, including women entrepreneurs, resource persons, family members, and representatives of support organizations. The final interviews primarily reinforced previously identified themes rather than introducing novel perspectives, indicating that sufficient depth and breadth of information had been obtained to address the research objectives.

### 3.4 Data analysis

The qualitative data were analyzed using Mucchielli (2006) content analysis approach (cited in Dany, 2016). This method provides a systematic framework for identifying, organizing, and interpreting patterns of meaning within qualitative data. The analysis was conducted in six sequential stages: (i) preparation of the corpus; (ii) definition of the units of analysis; (iii) coding and categorization; (iv) thematic analysis; (v) interpretation of the findings; and (vi) validation of the analytical results.

All interviews were transcribed verbatim and organized into a textual corpus. The transcripts were then segmented into sentence-based units of analysis to facilitate systematic coding. A hybrid coding strategy, combining deductive and inductive approaches, was employed. Initial codes were informed by the research objectives, the conceptual framework, and the interview guide, while additional codes emerged iteratively from the empirical data during the analytical process.

To enhance the consistency and transparency of the analysis, a structured codebook was developed and refined throughout the coding process. Coding decisions were reviewed through iterative revisions and regular discussions among the research team to ensure conceptual consistency and minimize interpretative bias. Themes were subsequently generated by grouping related codes according to their conceptual similarity, recurrence, and analytical relevance. In addition to identifying recurrent patterns across the dataset, the analysis considered both manifest content and latent content (underlying meanings and interpretations).

The credibility and trustworthiness of the findings were strengthened through the triangulation of data obtained from different participant groups and across the two study sites. The emerging themes were interpreted in relation to the existing literature and the study's conceptual framework, enabling a contextualized understanding of women's entrepreneurship in southern Benin. Methodological rigor was further enhanced through prolonged engagement in the field, systematic coding procedures, and an iterative analytical process, thereby strengthening the transparency, credibility, and dependability of the findings.

## 4 Results

The findings are presented in four interrelated parts: (i) the socio-demographic characteristics of the participants, (ii) the vulnerabilities experienced by women entrepreneurs, (iii) the strategies they developed to sustain and expand their businesses, and (iv) the social reception of these strategies within family and community settings. Taken together, the results show that women's entrepreneurship in southern Benin is shaped by intersecting social, economic, and relational constraints, but also by adaptive forms of agency.

### 4.1 Socio- demographic characteristics of participants

Table 1 summarizes the demographic and other relevant characteristics of the interview participants.

**Table 1:** Socio-demographic characteristics of the interview participants.

Variables	Characteristics	Frequency	Percent
Gender	Female	34	66.7
	Male	17	33.3
	**Total**	**51**	**100.0**
Age group	18–20	6	11.8
	21–30	7	13.7
	31–40	5	9.8
	41–50	23	45.1
	51 and over	10	19.6
	**Total**	**51**	**100.0**
Educational attainment	Primary education	13	25.5
	Lower secondary education	15	29.4
	Upper secondary education	13	25.5
	Tertiary education	4	7.8
	No formal education	6	11.8
	**Total**	**51**	**100.0**
Marital status	Single	9	17.6
	Married	36	70.6
	Divorced	6	11.8
	**Total**	**51**	**100.0**
Occupation	Public employees	5	9.8
	Private employees	7	13.7
	NGO workers	6	11.8
	Educators and trainers	3	5.9
	Taxi drivers	2	3.9
	Craft entrepreneurs	21	41.2
	Traders/resellers	7	13.7
	**Total**	**51**	**100.0**

Source: Own illustration of the author, 2026. Bold values indicate the total count and percentage for each variable.

The study involved 51 participants, including 34 women (66.7%) and 17 men (33.3%). This distribution reflects the study's focus on women's entrepreneurship while also incorporating the perspectives of institutional actors, family members, and other stakeholders. The age structure of the sample was dominated by middle-aged respondents: 45.1% were aged 41–50 years, while 19.6% were aged 51 years and above. Younger participants aged 18–30 years accounted for 25.5% of the sample. This configuration suggests that the study captured the views of individuals with substantial life experience and, in many cases, long exposure to entrepreneurial activity or support systems.

Educational attainment was generally modest. Nearly one-third of participants had completed lower secondary education (29.4%), while 25.5% had primary education and an equivalent proportion had upper secondary education. Only 7.8% had tertiary education, whereas 11.8% reported no formal schooling. This profile reflects the educational characteristics commonly observed among small-scale entrepreneurs in Benin and provides important context for understanding both the opportunities available to women and the constraints they face in structuring and expanding their businesses.

Marital status further highlights the centrality of family life in the participants' social worlds. Most respondents were married (70.6%), followed by single participants (17.6%) and divorced participants (11.8%). In a setting where household responsibilities, marital expectations, and kinship relations strongly shape women's economic participation, this distribution is analytically significant. Occupationally, the sample was diverse but remained concentrated in entrepreneurial and near-entrepreneurial activities. Craft entrepreneurs constituted the largest group (41.2%), followed by traders and resellers (13.7%). The remaining participants were private employees (13.7%), NGO workers (11.8%), public employees (9.8%), educators and trainers (5.9%), and taxi drivers (3.9%). This occupational diversity made it possible to capture a broad range of perceptions while maintaining a strong focus on women's business activities.

### 4.2 Vulnerabilities experienced by women entrepreneurs

The thematic analysis explored how participants perceived the vulnerabilities experienced by women entrepreneurs, with particular attention to the influence of gender and age. The analysis was guided by recurrent patterns identified across the interview data and resulted in three interconnected themes: (i) common gender-related vulnerabilities, (ii) age-specific vulnerabilities, and (iii) the main sectors in which women entrepreneurs operate. Together, these themes provide insight into the ways participants described the social and economic factors shaping women's entrepreneurial experiences.

#### 4.2.1 The common characteristics of vulnerability among women entrepreneurs

The thematic analysis identified gendered socio-cultural norms as a central theme emerging from participants' accounts of women's entrepreneurial experiences. This theme was developed from recurrent codes relating to traditional gender roles, division of labor, socialization practices, and patriarchal power relations. Across interviews, participants consistently described these interconnected factors as influencing women's access to entrepreneurial opportunities and shaping expectations regarding their economic and domestic responsibilities.

Participants commonly portrayed the socio-cultural context as one in which inequalities in professional roles and opportunities between men and women continue to persist. Entrepreneurship was frequently described as being socially associated with men, whereas women were generally expected to prioritize domestic and caregiving responsibilities. Several interviewees suggested that these perceptions continue to influence family expectations, community attitudes, and women's participation in entrepreneurial activities.

These perspectives were often illustrated through local cultural expressions. One participant referred to a well-known proverb in Fongbé, the most widely spoken language in southern Benin: “Sunu glegbenu, gnonu xesi,” which implies that men are responsible for agricultural production, whereas women are expected to remain within the domestic sphere. Similar references to traditional beliefs were made by participants from different stakeholder groups, suggesting that such cultural narratives continue to influence how entrepreneurial roles are perceived within many communities, despite participants also acknowledging gradual social change.

Participants further explained that these gendered expectations are reproduced through early socialization. According to several interviewees, boys are generally encouraged to engage in productive economic activities alongside their fathers or other male relatives, whereas girls are more frequently expected to undertake domestic responsibilities. Participants perceived these early experiences as shaping later educational aspirations, occupational choices, and opportunities to engage in entrepreneurship. Rather than viewing these constraints solely as individual challenges, many respondents described them as reflecting broader social and cultural expectations that influence women's entrepreneurial pathways.

The following quotation illustrates one participant's perspective, which was broadly consistent with views expressed across the interviews:

“Although some changes have emerged in relation to this cultural norm, which historically confines women to domestic roles, it continues to reproduce and legitimize unequal power relations between men and women, as well as between boys and girls and within marital relationships. In doing so, it also constrains women's access to and advancement within professional spheres, thereby limiting their entrepreneurial and career opportunities” (S. Y., Resource person from NGO, male, Abomey-Calavi, December 2025).

Although this quotation reflects the views of one participant, similar concerns were raised by several interviewees representing different stakeholder groups. Collectively, these accounts suggest that many participants perceived socio-cultural norms as continuing to influence women's access to entrepreneurial opportunities and decision-making within both household and professional settings. While participants also recognized that attitudes toward women's economic participation are evolving, they frequently described traditional gender expectations as remaining an important consideration in their entrepreneurial experiences.

The interview data suggest that participants viewed gender-related vulnerability as being closely associated with wider socio-cultural expectations rather than solely with individual circumstances. These findings indicate that, from the participants' perspective, efforts to strengthen women's entrepreneurship may benefit from addressing not only economic constraints but also the social and institutional norms that shape opportunities for women's participation in entrepreneurial activities.

#### 4.2.2 Specific vulnerabilities of female SMEs based on age

The thematic analysis identified age-related vulnerability as a distinct sub-theme within participants' accounts of women's entrepreneurial experiences. This sub-theme emerged from recurrent codes relating to life-course transitions, social legitimacy, marital status, household responsibilities, and decision-making power. Although participants consistently identified gender-related barriers affecting women entrepreneurs, the interviews suggested that these barriers were experienced differently according to age and stage of life.

Across the interviews, participants described younger women as occupying a relatively vulnerable position within a social context characterized by gerontocratic norms, where authority, credibility, and decision-making are often associated with older adults. Several respondents explained that younger women may encounter difficulties in gaining recognition for their entrepreneurial aspirations because they are sometimes perceived as lacking the experience or social standing required to establish and manage a business. As a result, many participants described young entrepreneurs as relying on encouragement and material assistance from family members, relatives, or partners during the start-up phase of their businesses.

This pattern is illustrated by the following participant:

“Few years ago, when I was in apprenticeship, my boyfriend used to help me. He also gave me advice on how I could later develop my own workshop. After completing my training, he supported me by buying a sewing machine and tools I needed to get started. That's how I set up my workshop, and it worked out well. I also started selling sewing materials alongside, which helped me diversify my income” (Mrs. F. D. 25 years, artisan, Abomey-Calavi, December 2025).

Although this quotation reflects one participant's experience, similar narratives were shared by several younger interviewees. Participants frequently highlighted the importance of financial, emotional, or technical support from relatives or partners in facilitating business creation. At the same time, some respondents suggested that reliance on external support could influence the degree of independence with which young women were able to make entrepreneurial decisions. These accounts indicate that, from the participants' perspective, the entrepreneurial experiences of younger women are shaped not only by financial constraints but also by their social position within family and community structures.

The interviews further suggested that women's entrepreneurial experiences evolve over the life course. Several participants perceived marriage as increasing women's social legitimacy within the community, while simultaneously introducing additional domestic responsibilities and expectations associated with household management. Married women commonly described the need to balance business activities with family obligations, particularly as their enterprises expanded.

Some participants also reported that business growth occasionally created tensions within marital relationships, particularly when increased entrepreneurial responsibilities appeared to conflict with traditional expectations regarding women's domestic roles. One entrepreneur described her experience as follows:

“Sometimes, I feel like my husband is jealous of my work. At one point, I had more than ten apprentices in my workshop, and during busy periods, we would work late into the night. He didn't approve of that—he felt I was prioritizing my business too much. Because of this, I had to reduce my workload, and my business suffered. He even told me he might take another wife to handle the household responsibilities. To protect my marriage, I eventually changed the way I managed my work” (Mrs. P. A., 46 years, hairstyle artisan, Abomey-Calavi, February 2025).

Although this quotation represents an individual account, comparable experiences were reported by several married participants. Interviewees described situations in which business expansion required negotiating competing expectations related to entrepreneurship and family responsibilities. Some participants perceived their entrepreneurial success as creating tension within existing household arrangements, while others explained that they voluntarily adjusted their working practices to maintain a balance between business commitments and family life. These accounts should therefore be understood as reflecting the experiences of the participants rather than suggesting that entrepreneurial growth inevitably leads to conflict within households.

Overall, the interview data suggest that participants perceived age and life stage as influencing the forms of vulnerability experienced by women entrepreneurs. Younger participants more frequently described challenges associated with limited recognition, restricted access to resources, and dependence on external support, whereas married and older participants more commonly discussed the complexities of balancing entrepreneurial aspirations with household responsibilities. These findings indicate that women's entrepreneurial experiences are shaped by changing social roles across the life course and suggest that support programmes may benefit from recognizing the differing needs and circumstances of women entrepreneurs at different stages of their personal and professional lives.

#### 4.2.3 Overview of main areas in which women operate

The thematic analysis identified sectoral concentration of women's entrepreneurial activities as a third theme. This theme emerged from recurrent codes relating to access to productive resources, occupational segregation, income-generating activities, and household livelihood strategies. Participants consistently described women's entrepreneurial activities as being concentrated in a limited number of sectors that were perceived to reflect both economic opportunities and prevailing socio-cultural expectations. Documentary evidence from national statistics was used to contextualize these accounts.

Women represented 51.2% of Benin's population in 2013 Institut National de la Statistique et de l‘Analyse Économique – INSAE, 2015 and 50.8% in 2020 (Banque Africaine de Développement – BA, 2021). Participants nevertheless perceived women's participation in the economy as being characterized by unequal access to productive resources and occupational opportunities. These perceptions are broadly consistent with national statistics showing that women account for a substantial proportion of the labor force, with participation across the primary (42.2%), secondary (18.1%), and tertiary (39.7%) sectors (Banque Africaine de Développement – BA, 2021). However, only 15.7% of agricultural households were headed by women in 2021 (Ministère de l'Agriculture, de l'Elevage et de la Pêche – MAEP, 2021), suggesting comparatively limited access to productive assets such as agricultural land.

Several participants attributed this situation to customary inheritance practices and long-standing socio-cultural norms that were perceived to restrict women's ownership and control of land. Interviewees suggested that these constraints may limit opportunities to expand agricultural enterprises and reduce access to financial resources that often depend on land ownership as collateral. Consequently, participants described many women as diversifying into commercial activities requiring relatively limited capital investment and fewer productive assets.

Small-scale trading emerged as one of the principal entrepreneurial activities discussed during the interviews. Participants frequently described local markets as providing accessible opportunities for women to generate income through the resale of agricultural produce, food products, and household commodities. Several interviewees explained that income generated from these activities was commonly used to meet both business and household needs, illustrating the close relationship between women's entrepreneurial activities and family livelihoods.

One participant described this experience as follows:

“I resell various goods, such as fresh tomatoes, rice and vegetable oil, as well as anything else you might need for cooking. Of course, I earn a small profit from this activity, but sometimes I use it to develop my business and often to cook for my family. Even if I receive a daily allowance from my husband, it is not really enough to cover our expenses” (Mrs. A. K, 49 years, Abomey-Calavi, February 2025).

Although this quotation represents one participant's experience, similar accounts were shared by several women engaged in petty trading. Participants commonly described their businesses as serving multiple purposes, including generating income, supporting household expenditure, and providing a degree of financial flexibility. Rather than portraying these enterprises solely as commercial ventures, interviewees frequently emphasized their contribution to household wellbeing and economic resilience.

The interviews also highlighted the importance of artisanal occupations as a source of women's entrepreneurship. Participants explained that many young women enter these activities through apprenticeship schemes, particularly those from economically disadvantaged households seeking vocational skills and self-employment opportunities. However, respondents consistently described occupational choices as being strongly influenced by gender norms. Hairdressing, dressmaking, and food processing were commonly identified as occupations considered socially appropriate for women, whereas mechanics, welding, carpentry, and metalwork continued to be perceived as predominantly male professions.

These accounts suggest that participants viewed women's concentration in particular sectors as reflecting a combination of economic necessity and socially constructed expectations regarding gender roles. While the interviews do not suggest that all women experience these constraints in the same way, they indicate that many participants perceived access to productive resources, occupational opportunities, and income-generating activities as being shaped by broader social and cultural factors. Overall, the findings suggest that initiatives aiming to strengthen women's entrepreneurship may benefit from addressing both resource inequalities and the gendered norms that influence occupational choices and business opportunities.

### 4.3 Strategic opportunities for women's businesses and challenges in family cohesion

Strategies that promote women's engagement in entrepreneurship encompass both financial and non-financial forms of support. The following section examines the ways in which women benefit from these diverse support mechanisms in the development and sustainability of their businesses.

#### 4.3.1 Access to financial support for women entrepreneurs

The thematic analysis identified access to financial support as a key theme influencing participants' entrepreneurial experiences. This theme emerged from recurrent codes relating to microfinance, savings requirements, informal finance, collective solidarity, and access to start-up capital. Across the interviews, participants consistently described access to finance as an important enabler of business creation and continuity, while also highlighting a number of practical constraints associated with existing financing mechanisms.

Participants frequently referred to decentralized financial institutions, commonly known as microcredit organizations, as one of the principal sources of financial support available to women entrepreneurs. These institutions were described as providing small and medium-sized loans to individuals engaged in income-generating activities, including artisans and small business owners. Several interviewees considered these services to be valuable in facilitating business establishment and day-to-day operations. However, participants also explained that eligibility for larger loans generally required applicants to accumulate prior savings, often amounting to approximately 50% of the requested loan value. According to several respondents, this requirement may present a significant obstacle for women with limited financial resources. Participants further noted that smaller loans of up to 25,000 XOF (approximately USD 45) could sometimes be obtained without collateral, thereby providing an accessible entry point for women seeking to initiate small-scale entrepreneurial activities.

Beyond formal financial institutions, participants consistently identified two complementary community-based financing mechanisms. The first comprised rotating savings and credit associations organized within artisans' organizations. Interviewees described these schemes as widely used because they are based on mutual trust, social solidarity, and reciprocal support among members, enabling participants to mobilize resources for business-related needs. The second mechanism involved informal financial intermediaries, commonly referred to as “*tontiniers*,” who were perceived as offering greater flexibility in responding to short-term financial needs than formal lending institutions.

Although participants generally viewed these financial arrangements positively, many also highlighted their limitations. Several interviewees suggested that the savings requirements imposed by formal microfinance institutions, together with the relatively modest size of available loans, could constrain opportunities for business expansion. Likewise, while informal financing mechanisms were often perceived as more accessible and responsive, some participants expressed concerns regarding their lack of formal regulation and the limited legal protection available to borrowers. These accounts indicate that participants viewed both formal and informal financing systems as offering valuable opportunities, while simultaneously presenting constraints that may affect women's capacity to develop their businesses.

Overall, the interview data suggest that access to finance remains an important, yet unevenly distributed, resource for women entrepreneurs. Participants described combining multiple financial strategies to establish and sustain their enterprises, reflecting both the opportunities and limitations of existing support mechanisms. These findings suggest that improving women's entrepreneurial development may require financial services that are not only more accessible but also better aligned with the varying needs and circumstances of women-owned businesses.

#### 4.3.2 Non-financial support for women entrepreneurs

The thematic analysis identified non-financial support as a second major theme shaping participants' entrepreneurial experiences. This theme emerged from recurrent codes relating to capacity building, material assistance, professional associations, knowledge sharing, and business development support. Across the interviews, participants consistently described non-financial support as complementing financial assistance by strengthening entrepreneurial skills, improving access to productive resources, and facilitating professional networking.

Participants frequently referred to professional organizations, development agencies, and public institutions as important providers of material and technical support. Several interviewees described receiving occupational equipment, vocational training, and mentoring through programmes implemented by international development partners, including the Swiss Agency for Development and Cooperation (SDC), the *Deutsche Gesellschaft für Internationale Zusammenarbeit* (GIZ), the World Bank, the United States Agency for International Development (USAID), the Danish International Development Agency (DANIDA), and national non-governmental organizations. Participants generally perceived these interventions as contributing to the development of technical competencies and supporting the establishment or improvement of their business activities.

One participant explained:

“We have received a lot of support from different intervention projects that help artisans develop their businesses. In my case, I was given hairstyling tools, which were also used during the training we received to improve and refine our work” (Mrs. A. M. 52 years, artisan, hairstyling, Cotonou, January 2026).

Although this quotation reflects one participant's experience, similar accounts were reported across the interviews. Participants commonly described material assistance as being most beneficial when combined with practical training and opportunities to apply newly acquired skills. Rather than viewing equipment or training as isolated interventions, many respondents perceived the combination of these forms of support as strengthening their confidence, improving service quality, and facilitating business development.

The interviews further highlighted the continuing role of artisans' associations in sustaining learning beyond externally funded development projects. Participants explained that these organizations have historically served as platforms through which artisans collectively address professional challenges, exchange knowledge, and organize mutual support. Several respondents indicated that, following the completion of donor-funded projects, many associations continued to organize regular training activities independently, thereby maintaining opportunities for professional development.

One participant described these activities as follows:

“We meet twice a week in our association. The first meeting, held every Wednesday, is dedicated to learning new hairstyling skills. As you know, things are constantly evolving, so we need to keep up with new styles. That is why we organize these training sessions regularly. The second meeting is devoted every Friday to our solidarity-based savings and loan activities within the association” (Mrs. E. T., 49 years, artisan, hairstyling, Abomey-Calavi, October 2024).

Comparable experiences were described by participants from several artisans' organizations. Interviewees consistently portrayed these associations as providing more than technical training. They were also viewed as spaces for peer learning, mutual encouragement, and collective problem-solving, while simultaneously facilitating access to solidarity-based savings and lending mechanisms. These accounts suggest that participants perceived local professional associations as making an important contribution to both entrepreneurial learning and business continuity.

Participants also discussed the role of entrepreneurship promotion policies and business development services. Several interviewees observed that entrepreneurship is increasingly encouraged within formal education and apprenticeship systems, contributing to growing interest in self-employment. However, respondents frequently identified limited knowledge of business planning, market analysis, and enterprise management as significant challenges during business creation. Although specialized support was reported to be available through consultants, private firms, and non-governmental organizations, participants often perceived these services as financially inaccessible, particularly for women operating with limited resources. Consequently, some interviewees described establishing businesses without formal business plans or strategic guidance, which they believed could affect subsequent business development.

Overall, the interview data suggest that participants viewed non-financial support as an important complement to financial assistance. Rather than relying solely on external development projects, many respondents emphasized the continuing value of locally organized initiatives within artisans' associations, particularly those combining skills development, peer learning, and solidarity-based financial support. At the same time, participants indicated that access to specialized business development services remained uneven, suggesting that strengthening affordable advisory and mentoring services could further enhance women's entrepreneurial opportunities. These findings should be interpreted as reflecting the experiences and perceptions reported by participants rather than suggesting that all women entrepreneurs encounter similar forms of support or constraints.

### 4.4 Strategies developed and their social reception in the entrepreneurs' trajectories

#### 4.4.1 Strategies developed by women

The thematic analysis identified adaptive entrepreneurial strategies as a major theme describing how participants sought to sustain and develop their businesses despite the social and economic constraints discussed previously. This theme emerged from recurrent codes relating to mobility, professional networking, decision-making autonomy, leadership, and financial management. Across the interviews, participants described adopting a range of strategies to strengthen their entrepreneurial activities, while also reflecting on how these strategies were perceived within their families and communities.

One recurring pattern concerned participation in activities that required women to spend time outside their homes or communities. Participants referred to attending vocational training, participating in professional meetings, traveling to markets, and engaging with external organizations as important opportunities for developing new skills and expanding business networks. However, several interviewees also reported that these activities could generate concerns among family members, particularly when they involved prolonged absences from home or reduced opportunities for spouses to oversee women's daily activities.

One participant described her experience as follows:

“I had the opportunity to participate in a training session on food processing in Cotonou for several weeks. When I discussed it with my husband, he immediately disagreed. However, I did not take his opinion into account and decided to attend the training. When I returned, my husband and his family decided to proceed with a divorce” (Mrs. R. I., 32 years, artisan, hairstyling, Cotonou, December 2025).

Although this quotation reflects an individual experience, comparable accounts were reported by several participants who described negotiating tensions between professional opportunities and family expectations. Participants suggested that activities involving increased mobility or greater engagement in public spaces were, in some instances, perceived as inconsistent with prevailing expectations regarding women's domestic roles. These accounts indicate that, for some women, pursuing entrepreneurial opportunities required negotiating complex family and social relationships, although such experiences were not reported by all participants.

Participants also discussed the interpersonal dynamics associated with accessing entrepreneurial support. Several interviewees noted that training programmes, financial services, and business support initiatives often required interaction with male trainers, advisers, or institutional representatives. While many participants viewed these interactions as a normal aspect of business development, some reported that they could occasionally give rise to misunderstanding or suspicion within their social environment. Participants therefore suggested that the organization of entrepreneurial support programmes should take greater account of the social contexts within which women operate.

A further sub-theme related to women's decision-making autonomy within their businesses. Several participants explained that managing enterprises independently, making investment decisions, or expanding business activities sometimes required negotiating established household expectations. Some interviewees perceived that increased economic independence could alter existing patterns of decision-making within the family, occasionally creating tension regarding household roles and responsibilities. Participants' accounts nevertheless varied considerably, indicating that these experiences depended on individual family circumstances rather than representing a universal feature of women's entrepreneurship.

Similar perspectives emerged in relation to women's growing participation in leadership roles within professional associations and community organizations. Several interviewees viewed these positions as valuable opportunities to strengthen professional networks, acquire leadership skills, and increase visibility within their sectors. At the same time, some participants perceived that greater public recognition could occasionally challenge traditional expectations regarding women's social roles. These observations suggest that participants experienced leadership as both an opportunity for professional development and, in some cases, a source of additional social negotiation.

Participants also reflected on the management of business income and household expenditure. Several interviewees explained that balancing business reinvestment with financial contributions to household needs represented an ongoing challenge. Some participants reported attempting to preserve business income for investment purposes while simultaneously responding to family expectations regarding household expenditure. One resource person explained:

“If you want to succeed in your business, it is important to know how to manage money properly. It is not advisable to use business resources for household expenses. In many cases, men tend to take advantage of women's business income. However, this practice poses a significant risk to the sustainability of the business…” (M. K. S., 55 years, resource person, NGO, Abomey-Calavi, December 2025).

Although this quotation reflects the perspective of one resource person, similar concerns were raised by several participants. Interviewees frequently emphasized the importance of separating household expenditure from business finances in order to maintain business continuity. At the same time, participants recognized that achieving this separation was not always straightforward, particularly where household economic needs were substantial. These accounts suggest that financial decision-making within women-owned businesses is often shaped by broader family circumstances and competing economic priorities.

Overall, the interview data suggest that participants viewed entrepreneurial development as involving continuous negotiation between business aspirations, family responsibilities, and prevailing social expectations. While many participants regarded the strategies they adopted as important for strengthening their businesses, some also described encountering social or familial challenges associated with these strategies. These findings should therefore be understood as reflecting the diverse experiences reported by participants rather than suggesting that entrepreneurial development inevitably generates family conflict or challenges established gender relations in all contexts.

#### 4.4.2 Social reception of entrepreneurial strategies

The thematic analysis identified social reception and negotiation of entrepreneurial roles as a major theme. This theme emerged from recurrent codes relating to gender expectations, family approval, social reputation, marital tensions, and community perceptions. Across the interviews, participants described a range of reactions to women's entrepreneurial activities, suggesting that the social reception of these strategies was often complex and varied according to family circumstances, community norms, and the nature of the business activity.

Several interviewees described difficulties in balancing entrepreneurial responsibilities with expectations associated with marriage, motherhood, and domestic roles. Participants suggested that, in some families and communities, women's professional ambitions were sometimes viewed as secondary to their reproductive and caregiving responsibilities. One resource person reflected on these perceptions as follows:

“I got the impression that most women who become owners of large-scale enterprises are unmarried or divorced due to the incompatibility of work and family life, and the social expectations placed on women in society. A woman entrepreneur is often under pressure from her own family, who consider marriage, giving birth and taking care of her family to be the most important things for a woman. This makes it seem like entrepreneurship is a male-dominated activity. And everyone acts as if this were true, including young people. I hope that we will remain undeveloped in this respect while social and cultural expectations continue to position women in this way” (M. R. Z., 65 years old, resource person and project manager, Cotonou, January 2026).

This quotation reflects the participant's perception that women's entrepreneurial ambitions may sometimes be viewed as difficult to reconcile with prevailing social expectations surrounding marriage and family life. Similar concerns were raised by other interviewees, who described experiencing pressure from relatives or community members to prioritize domestic responsibilities. These accounts suggest that some participants perceived entrepreneurship as continuing to be socially associated with men, although the interviews also revealed examples of women who were actively pursuing entrepreneurial careers while maintaining family responsibilities. The findings should therefore be interpreted as reflecting participants' experiences and perceptions rather than as evidence that entrepreneurship is inherently incompatible with family life.

Participants also discussed the potential impact of negative social perceptions on women's confidence and entrepreneurial aspirations. Some interviewees reported that women who spent considerable time in public or professional spaces could be labeled as neglecting their domestic responsibilities or behaving in ways considered socially inappropriate. A number of respondents referred to stigmatizing expressions used within their communities, including labels such as “*irresponsible mother*” or “*unfaithful wife*.”

However, participants' accounts varied, and not all women reported experiencing such reactions.

Several interviewees further described situations in which entrepreneurial activities became a source of disagreement within households. Participants mentioned experiences such as disputes over time allocation, concerns about business-related travel, or disagreements regarding the use of business income. Some women reported receiving reduced financial or emotional support from their spouses, while others described negotiating new arrangements to accommodate both family and business responsibilities. These accounts indicate that entrepreneurial activities could, in some circumstances, become one of several factors contributing to household tension, although the interviews do not suggest that such outcomes were universal.

Participants also reflected on how these dynamics could affect relationships with extended family members and the wider community. In some cases, interviewees perceived that relatives became involved in disputes relating to women's entrepreneurial activities, which could either intensify tensions or facilitate mediation. A few participants described periods of marital separation or reduced cooperation with their spouses, whereas others reported that initial resistance diminished over time as their businesses became more established. These differing experiences highlight the diversity of women's entrepreneurial trajectories and caution against assuming a single pattern of social reception.

A family resource person offered the following perspective:

“Women are generally reduced to the household and are not suited to large enterprises. Our customs impose many restrictions on women. We are essentially born with limited opportunities to become brave, unconstrained women. I don't believe that, in our context, women will become very independent. It's a false promise” (Mrs O. A., 54, family resource person, Cotonou, February 2026).

This statement illustrates the persistence of restrictive gender norms within some segments of society. Although it represents the opinion of one participant, comparable views were expressed by several interviewees who described cultural expectations as limiting women's opportunities and shaping their aspirations. At the same time, other participants challenged these assumptions by emphasizing women's capacity to manage businesses successfully and contribute to household and community wellbeing. The coexistence of these contrasting perspectives suggests that social attitudes toward women's entrepreneurship are neither uniform nor static.

Younger women were often described as being subject to closer scrutiny regarding their mobility, social relationships, and business activities. Some participants perceived that young women's entrepreneurial initiatives were more likely to be questioned by family members, particularly before marriage. However, experiences varied considerably, with some younger entrepreneurs reporting strong support from relatives and mentors. These findings therefore indicate that age may influence how entrepreneurial activities are socially interpreted, but they do not suggest that all young women experience the same level of resistance.

The interview data suggest that women's entrepreneurial strategies were received in different ways across households and communities. Many participants described entrepreneurship as creating opportunities for economic advancement and personal development, while some also reported encountering social criticism, family pressure, or challenges in negotiating changing roles and responsibilities. The findings therefore point to the importance of understanding women's entrepreneurship as a socially negotiated process, shaped by diverse family and community contexts rather than by a single or inevitable pattern of conflict or exclusion.

## 5 Discussion

Drawing on social role theory (Eagly, 1987; Ridgeway, 2011) and strategic actor theory (Crozier and Friedberg, 1977), this study provides insights into how women entrepreneurs in southern Benin perceive and negotiate the opportunities and constraints shaping their entrepreneurial trajectories. The discussion focuses on two interrelated issues: (i) structural vulnerability and intersectional constraints, and (ii) women's strategic agency in managing entrepreneurship within family and community settings.

### 5.1 Structural vulnerability and intersectional constraints in women entrepreneurship

Drawing on social role theory (Eagly, 1987; Ridgeway, 2011), the interview data suggest that many participants perceived women's entrepreneurial activities as being shaped by socially prescribed gender roles that continue to influence access to economic opportunities, productive resources and decision-making. Participants frequently described entrepreneurship as developing within a social context where men are generally expected to assume the role of economic providers, while women are expected to prioritize domestic and caregiving responsibilities. These perceptions are consistent with previous studies showing that gendered social expectations continue to shape entrepreneurial opportunities and constraints (Conteh et al., 2021; Grönlund and Oun, 2018).

The findings also suggest that women's entrepreneurial experiences are not homogeneous but vary according to age and life stage. Participants described younger women as facing challenges associated with limited social recognition and restricted access to resources, whereas married and older women more frequently discussed negotiating entrepreneurial aspirations alongside household and family responsibilities. These accounts support previous research highlighting the intersection between gender and other social characteristics, including age and family status, in shaping entrepreneurial experiences (Nsabiyumva et al., 2022; Gerard et al., 2025; Tunberg and Anderson, 2020). Rather than suggesting a universal pattern, the present findings indicate that participants perceived entrepreneurial vulnerability as evolving across the life course and being influenced by the interaction of gender, age and family circumstances.

The findings reinforce the argument that women's entrepreneurial trajectories are socially embedded and negotiated within broader family and community contexts (Amubode et al., 2016; García Escribano and Casado, 2016). However, the diversity of participants' experiences also suggests that the influence of socio-cultural norms is neither fixed nor uniform. While several interviewees described significant constraints, others reported supportive family relationships and opportunities to negotiate existing gender expectations.

### 5.2 Strategic agency and reconfiguration of entrepreneurship within the family

From the perspective of strategic actor theory (Crozier and Friedberg, 1977), the findings show that women do not simply endure structural constraints but actively develop strategies to expand their entrepreneurial possibilities. Participants described mobilizing support from both formal and informal organizations, participating in professional associations, attending vocational training, and using community-based financial arrangements to establish or expand their businesses. These practices demonstrate that women exercise agency within existing structures, using available resources to create room for maneuver in constrained environments.

At the same time, the findings reveal that access to such opportunities remains uneven. Financial support was often small in scale and conditional upon prior savings, while specialized business development services were frequently perceived as too costly for many women. As a result, participants commonly relied on combinations of formal and informal mechanisms to sustain their activities. These observations align with earlier studies highlighting the importance of institutional and social support for women's entrepreneurship (Muhammad and Ximei, 2022; Franzke et al., 2022). They also suggest that support systems are most effective when they are adapted to women's material realities rather than designed as one-size-fits-all solutions.

The interviews further indicate that entrepreneurial development often requires ongoing negotiation between business aspirations and family expectations. Some participants reported that economic independence, mobility, and leadership responsibilities could create tension within the household or broader community. Others, however, described receiving encouragement from spouses, relatives, or professional networks. These contrasting experiences suggest that family relations can function as both enabling and constraining forces, depending on the circumstances. Accordingly, the findings do not imply that entrepreneurship inevitably threatens family cohesion; rather, they show that women continually negotiate the terms under which entrepreneurship becomes socially acceptable and personally sustainable.

Taken together, the findings highlight women's entrepreneurial agency while recognizing the continuing influence of gendered social expectations. Rather than portraying women solely as constrained by structural inequalities, the study illustrates how participants actively negotiated opportunities, mobilized resources and adapted their entrepreneurial strategies within changing family and institutional contexts.

## 6 Limitations of the research

Although this study offers important qualitative insights into women's entrepreneurial experiences in southern Benin, several limitations should be acknowledged. First, the research was conducted in Cotonou and Abomey-Calavi, two major urban centers, and therefore does not capture the full diversity of entrepreneurial experiences across other regions of Benin. Second, because the study is qualitative, the findings are intended to provide analytical rather than statistical generalization. Third, the data are based on self-reported interviews, which may be influenced by recall, interpretation, or social desirability bias. For these reasons, the results should be understood as reflecting the experiences and perceptions of the participants interviewed, rather than as representative of all women entrepreneurs in Benin.

## 7 Conclusion

In the contemporary context of global initiatives promoting gender equality and women's empowerment, this qualitative research contributes to ongoing efforts toward the achievement of Sustainable Development Goal 5. Conducted in Cotonou and Abomey-Calavi, two major economic centers in southern Benin, the study explored the entrepreneurial experiences of women operating in the craft sector. Data were collected from 51 participants, including young and adult women entrepreneurs, as well as representatives from public institutions, private organizations, non-governmental organizations involved in supporting women's entrepreneurship, and family members.

The findings reveal that women's entrepreneurial trajectories continue to be shaped by deeply embedded socio-cultural norms influencing their economic roles, access to opportunities, and investment capacities. Participants' accounts indicate that gendered expectations remain important factors contributing to women's vulnerability within entrepreneurial contexts. At the same time, the study highlights the contribution of various financial and non-financial support mechanisms implemented by international agencies and national actors in strengthening women's entrepreneurial capacities.

However, the strategies developed by women to sustain and expand their businesses are often negotiated within existing social and familial structures. Participants described how entrepreneurial initiatives may sometimes create tensions with prevailing expectations regarding women's domestic and family responsibilities. These findings highlight the intersectional nature of women's entrepreneurial vulnerability, particularly through the interaction of gender, age, and family-related dynamics. At the same time, they demonstrate women's capacity for strategic agency through their ability to mobilize resources, develop adaptive strategies, and pursue entrepreneurial opportunities despite the constraints they encounter.

This research contributes to knowledge by showing that women's entrepreneurship in southern Benin is not only an economic activity but also a socially embedded process shaped by gender relations, age-related inequalities, and family power dynamics. The findings suggest that strengthening women's entrepreneurship requires addressing not only access to financial and technical support but also the broader socio-cultural conditions that influence women's ability to develop and sustain their businesses. Therefore, reducing structural barriers requires coordinated efforts involving public policies, institutional interventions, and gender-sensitive education reforms aimed at promoting more inclusive and sustainable entrepreneurial pathways for women in Benin.

Future research could further investigate how traditional norms and cultural values may be transformed or mobilized to support women's empowerment and contribute to more equitable entrepreneurial environments within Benin.

## Data Availability

The original contributions presented in the study are included in the article/supplementary material, further inquiries can be directed to the corresponding author.
